# Microvascular Fragments Protect Ischemic Musculocutaneous Flap Tissue from Necrosis by Improving Nutritive Tissue Perfusion and Suppressing Apoptosis

**DOI:** 10.3390/biomedicines11051454

**Published:** 2023-05-16

**Authors:** Andrea Weinzierl, Yves Harder, Daniel Schmauss, Michael D. Menger, Matthias W. Laschke

**Affiliations:** 1 Institute for Clinical & Experimental Surgery, Saarland University, 66421 Homburg/Saar, Germany; 2 Department of Plastic Surgery and Hand Surgery, University Hospital Zurich, 8091 Zurich, Switzerland; 3 Department of Plastic, Reconstructive and Aesthetic Surgery, Ospedale Regionale di Lugano, Ente Ospedaliero Cantonale (EOC), 6900 Lugano, Switzerland; 4 Faculty of Biomedical Sciences, Università della Svizzera Italiana, 6900 Lugano, Switzerland

**Keywords:** microvascular fragments, tissue engineering, random-pattern flap, necrosis, apoptosis, angiogenesis, microcirculation

## Abstract

Microvascular fragments (MVF) derived from enzymatically digested adipose tissue are functional vessel segments that have been shown to increase the survival rate of surgical flaps. However, the underlying mechanisms have not been clarified so far. To achieve this, we raised random-pattern musculocutaneous flaps on the back of wild-type mice and mounted them into dorsal skinfold chambers. The flaps were injected with MVF that were freshly isolated from green fluorescent protein-positive (GFP^+^) donor mice or saline solution (control). On days 1, 3, 5, 7, and 10 after surgery, intravital fluorescence microscopy was performed for the quantitative assessment of angiogenesis, nutritive blood perfusion, and flap necrosis. Subsequently, the flaps were analyzed by histology and immunohistochemistry. The injection of MVF reduced necrosis of the ischemic flap tissue by ~20%. When compared to controls, MVF-injected flaps also displayed a significantly higher functional capillary density and number of newly formed microvessels in the transition zone, where vital tissue bordered on necrotic tissue. Immunohistochemical analyses revealed a markedly lower number of cleaved caspase-3^+^ apoptotic cells in the transition zone of MVF-injected flaps and a significantly increased number of CD31^+^ microvessels in both the flaps’ base and transition zone. Up to ~10% of these microvessels were GFP^+^, proving their origin from injected MVF. These findings demonstrate that MVF reduce flap necrosis by increasing angiogenesis, improving nutritive tissue perfusion, and suppressing apoptosis. Hence, the injection of MVF may represent a promising strategy to reduce ischemia-induced flap necrosis in future clinical practice.

## 1. Introduction

Random-pattern flaps are frequently used to reconstruct tissue defects in plastic surgery. However, the length-to-width ratio of such flaps cannot exceed certain values, because otherwise distal flap areas become vulnerable to necrosis. This is due to an inadequate blood perfusion of the tissue, which causes oxidative stress and inflammation, as well as ischemic and apoptotic cell death [1,2]. Accordingly, maintaining and boosting tissue vascularization is vital to ensuring an optimal outcome in flap surgery [3]. A promising approach to achieve this goal is the use of adipose tissue-derived microvascular fragments (MVF) [4,5].

MVF are functional arteriolar, capillary, and venular vessel segments that can be isolated from adipose tissue using mechanical dissection and short-term enzymatic digestion [6]. These fragments exhibit a length of up to 150–200 µm and an intact vessel morphology consisting of a central lumen with surrounding endothelial cells and stabilizing pericytes [7]. Moreover, they contain mesenchymal stem cells (MSC) within their physiological niche [8] and are a rich source of angiogenic growth factors, such as vascular endothelial growth factor (VEGF) and basic fibroblast growth factor (bFGF) [9,10]. Finally, MVF rapidly reform new blood-perfused microvascular networks after transplantation into tissue defects [11,12,13]. 

All these beneficial properties make the use of MVF a promising approach for vascularization with various applications in the field of tissue engineering and regenerative medicine [10,14,15]. In this context, they have also been shown to prevent necrosis of random-pattern skin flaps in rats [4,5]. However, the underlying mechanisms remain incompletely understood. Nakano et al. [5] speculated that the improved survival of the distal part of the flaps may be caused by early patent connections between transplanted MVF and the host microvasculature. Contrary to this assumption, Stone and Rathbone [4] did not detect a difference in the microvessel density between MVF-injected flaps and controls. Hence, they hypothesized that the increased flap survival may be rather promoted by MVF-associated stem cells.

To better understand the beneficial effects of MVF on flap tissue survival, we herein injected freshly isolated MVF from green fluorescent protein-positive (GFP^+^) donor mice or saline solution (control) into random-pattern musculocutaneous flaps, which were mounted into the dorsal skinfold chamber of GFP^−^ wild-type mice. This GFP^+^/GFP^−^ cross-over design allowed us to clarify in detail the fate of MVF after transplantation and to quantitatively assess angiogenesis, nutritive blood perfusion, inflammation, apoptosis, and necrosis of the flap tissue over a 10-day observation period by means of repeated intravital fluorescence microscopy as well as histology and immunohistochemistry.

## 2. Materials and Methods

### 2.1. Animals 

All animal experiments were approved by the local governmental animal protection committee (permit number: 10/2020). The study was executed according to the European legislation on the protection of animals (Directive 2010/63/EU) and the NIH Guidelines on the Care and Use of Laboratory Animals (NIH publication #85-23 Rev. 1985).

A total of 24 mice were used for the present study. Sixteen animals were C57BL/6 wild-type mice (Institute for Clinical and Experimental Surgery, Saarland University, Homburg/Saar, Germany), and eight mice expressed GFP (C57BL/6-Tg (CAG-EGFP)1Osb/J; The Jackson Laboratory, Bar Harbor, ME, USA). GFP^+^ animals were used for the experiments at an age of 30–52 weeks and a body weight of 30–35 g to serve as fat donors for the isolation of MVF. The remaining 16 animals exhibited an age of 12–24 weeks and a body weight of 26–30 g. On each animal, a random-pattern musculocutaneous flap was raised on the back and fixated within a dorsal skinfold chamber. The chamber-bearing animals were housed in individual cages at a room temperature of 22–24 °C, a relative humidity of 50–55%, and a 12 h day/night cycle for the duration of the experiments. The animals had unrestricted access to standard pellet chow (Altromin, Lage, Germany) and tap water.

### 2.2. MVF Isolation

**Figure 1:**
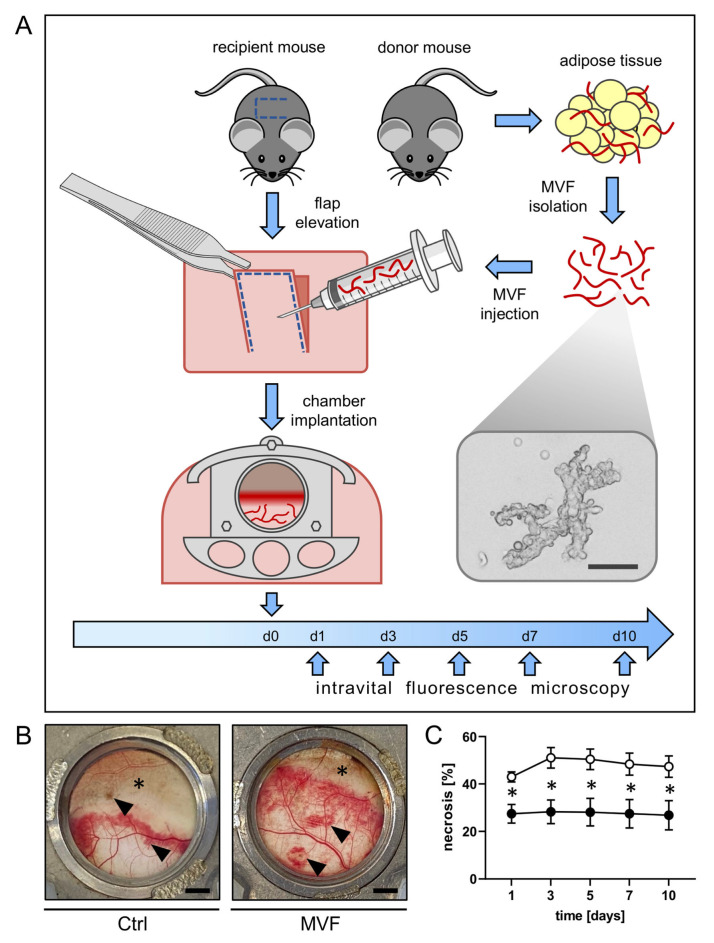
(**A**) Schematic depiction of the experimental setting. On day 0, a random-pattern flap was elevated on the back of a GFP^−^ wild-type recipient mouse. Microvascular fragments (MVF; inset = microscopic image of a single MVF; scale bar: 20 μm) isolated from the epididymal fat pads of a GFP^+^ donor mouse were injected into the flap and the dorsal skinfold chamber implantation was completed. Subsequently, repeated intravital fluorescence microscopy of the flap tissue was conducted on days 1, 3, 5, 7, and 10. (**B**) Macroscopic images of the chamber observation windows of a vehicle-injected control mouse (Ctrl) and an MVF-injected animal (MVF) on day 5 after flap elevation. The distal flap tissue has undergone necrosis (marked by asterisks), and the injection sites have remained visible (marked by arrowheads). Scale bar: 2 mm. (**C**) Necrosis [%] of flaps in MVF-injected mice (black circles, *n* = 8) and vehicle-injected controls (white circles, *n* = 8) on days 1, 3, 5, 7, and 10 after flap elevation, as assessed by intravital fluorescence microscopy and computer-assisted image analysis. Means ± SEM. * *p* < 0.05 vs. control.

MVF were isolated from the epididymal fat pads of male GFP^+^ donor mice, as previously described in detail [6] (Figure 1A). The epididymal fat pads on both sides were surgically removed and placed into 10% Dulbecco’s Modified Eagle’s Medium (DMEM; 100 U/mL penicillin, 0.1 mg/mL streptomycin; Biochrom, Berlin, Germany). The fat tissue was then cleaned by rinsing it three times in phosphate-buffered saline (PBS). The excised fat tissue was then diced into small pieces and digested with the enzyme collagenase NB4G (0.5 U/mL; Serva Heidelberg, Germany) under gentle agitation and high humidity (37 °C, 5% CO_2_) for 10 min. The digestion process was interrupted by neutralization with PBS containing 20% fetal calf serum (FCS), and the suspension of cells and vessels was subsequently incubated for 5 min at 37 °C. After the supernatant of floating fat was removed, the remaining suspension was strained through a 300 µm filter, and the MVF were condensed to a pellet by a 5 min centrifugation at 120× *g*. The MVF pellet was suspended once again in 10 µL 0.9% NaCl for flap injection. 

In line with previous studies [16], ~40.000 MVF could be isolated from each donor animal. For the present study, each flap was injected with the MVF from a single donor animal, as suggested by Nakano et al. [5].

### 2.3. Anesthesia

The surgical flap elevation and dorsal skinfold chamber implantation as well as the subsequent intravital fluorescent microscopic analyses were performed under general anesthesia by means of intraperitoneal injection of ketamine (100 mg/kg body weight; Ursotamin^®^; Serumwerke Bernburg, Bernburg, Germany) and xylazine (12 mg/kg body weight; Rompun^®^; Bayer, Leverkusen, Germany). After the chamber implantation, all animals received a subcutaneous injection of buprenorphine hydrochloride (0.01 mg/kg body weight; Temgesic^®^; RB Pharmaceuticals Limited, Slough, UK) to prevent postoperative pain.

### 2.4. Dorsal Skinfold Chamber

A flap was raised on the back of each animal and mounted into a dorsal skinfold chamber (Irola Industriekomponenten GmbH & Co. KG, Schonach, Germany) [17]. This flap is termed musculocutaneous, as mouse skin contains a thin muscle layer, the so-called panniculus carnosus muscle [17]. Our approach permitted repeated intravital fluorescence microscopy to study the microcirculation within the flap tissue. For the preparation, the anesthetized animal was depilated. Afterwards, a random-pattern musculocutaneous flap measuring 15 mm (width) × 11 mm (length) was elevated at a right angle to the spine on the back of each animal. The lateral flap margins were sutured back to the wound bed. The dorsal skinfold and the attached musculocutaneous flap were then sutured to the superior margin of one chamber frame. Adhesive insulation foam was used to cover the second chamber frame to ensure an air-tight chamber. The second frame was then mounted to its counterpart with screws. The flap was thus secured in between the two frames and was accessible for microscopic imaging through the chamber’s observation window. 

Within the chamber window, each flap was injected with ~40.000 MVF from one donor animal suspended in 10 µL saline solution (*n* = 8) or vehicle alone (*n* = 8) and distributed into 10 evenly dispersed sites. Injection was performed underneath the panniculus carnosus muscle into the subcutis, as injection into this space is easily reproducible while allowing high-quality visualization by means of intravital fluorescence microscopy [18]. A cover glass secured with a snap ring was used to close the observation window. Due to its conformation with a standardized width-to-length ratio, the flap developed ~50% necrosis without treatment as a result of the acute persistent ischemia (Figure 1B) [17,19].

After surgery, there was a 24 h recovery period for the animals before the first microscopy was performed. All animals showed normal feeding and sleeping patterns during the observation period without any signs of distress. After the last microscopy, the animals were killed by cervical dislocation under general anesthesia. Subsequently, the flap tissue was harvested for histological and immunohistochemical analysis.

### 2.5. Intravital Fluorescence Microscopy 

Intravital fluorescence microscopy was performed on days 1, 3, 5, 7, and 10 after flap elevation. The anesthetized mice were secured on a platform made of plexiglas. To enhance contrast between the blood vessels and the surrounding tissue, the mice received 0.1 mL of 5% fluorescein isothiocyanate (FITC)-labeled dextran (150,000 Da; Sigma-Aldrich, Taufkirchen, Germany) as a blood plasma marker by injection into the retrobulbar venous plexus. The chambers of MVF-injected mice were additionally scanned and recorded before the injection of the fluorescence dye to detect the GFP signal of viable MVFs. A Zeiss Axiotech fluorescence epi-illumination microscope (Zeiss, Oberkochen, Germany) was used to perform the intravital microscopies. During each microscopy, the microcirculation of the flap tissue was recorded on DVD for offline analysis. All microscopies were performed at a constant room temperature of ~22 °C. 

At the beginning of each microscopy, a panoramic view of the chamber was recorded for planimetric measurement of the perfused tissue surface. Within each flap, three observational zones were created by subdividing the flap into a proximal, medial, and distal zone. Two regions of interest (ROI) were selected per zone, each containing an arterio-venous bundle. Due to the distinct morphology of the recorded bundles, they could be identified during each microscopy for repeated measurements. Moreover, two capillary fields were recorded in the surrounding tissue of each selected arterio-venous bundle. If an ROI was no longer perfused, the bundle was documented by means of microscopic images throughout the rest of the observation period or as long as it could be identified. Within the medial transition zone between perfused and non-perfused tissue, an additional ROI was recorded to examine the formation of new microvessels. 

Microcirculatory parameters were assessed using CapImage (Version 8.5, Zeintl, Heidelberg, Germany) as an offline analysis system. The necrosis rate [%] was determined as 100-perfused surface area/total chamber surface area × 100. The functional capillary density (FCD) [cm/cm^2^] was determined as the total length of all perfused capillaries per capillary field. Within the proximal, medial, and distal ROI, microhemodynamic parameters were assessed in arterioles, capillaries, and venules. Vessel diameters (D) [µm] were measured at a right angle to the vessel path. The line shift method was used to measure centerline red blood cell (RBC) velocity (v) [mm/s] [20]. The volumetric blood flow (VQ) [pL/s] was then calculated using parameters v and D as $\mathrm{VQ}=\pi \times {\left(\frac{D}{2}\right)}^{2}\times \frac{v}{K}\;$ where K (=1.6) is the Baker–Wayland factor accounting for the parabolic velocity profile of blood in microvessels [21]. Angiogenesis within the transition zone was assessed by quantifying the density of neovessels [cm/cm^2^], which were identified by their irregular and entangled conformation that clearly differs from the straight, parallelly arranged capillaries of the panniculus carnosus muscle [22].

### 2.6. Histology and Immunohistochemistry

The obtained tissue samples of flap tissue were fixed using formalin, embedded in paraffin, and subsequently cut into 3 µm-thick sections. For the initial evaluation of the tissue, hematoxylin and eosin (HE) staining of individual sections was performed according to standard protocol. A BX60 microscope (Olympus, Hamburg, Germany) and the imaging software cellSens Dimension 1.11 (Olympus) were used for tissue assessment.

For the immunohistochemical identification of microvessels, sections were stained with a monoclonal rat anti-mouse CD31 antibody (1:100; Dianova, Hamburg, Germany) as the first antibody. A goat anti-rat Alexa 555 antibody (1:100; Invitrogen, Waltham, MA, USA) was used as the second antibody. The sections were then stained with a polyclonal goat GFP antibody (1:100; Rockland Immunochemicals Inc., Limerick, DE, USA) and a donkey-anti-goat biotin-labeled antibody (1:100; Life Technologies, Carlsbad, CA, USA) as well as Alexa 488-labeled streptavidin (1:50; Invitrogen). Using this staining, the origin of the contained microvessels could be determined. The nuclei of the cells were stained with Hoechst 33342 (2 μL/mL; Sigma-Aldrich) to superimpose images exactly. The stained sections were used to analyze microvessel density (all CD31^+^ microvessels per high-power field (HPF)). Furthermore, the fraction of CD31^+^/GFP^+^ microvessels [in %] was determined in two randomly chosen HPF at the flap base (proximal zone) and in the medial transition zone, where vital tissue bordered on necrotic tissue.

To identify myeloperoxidase-positive (MPO^+^) neutrophilic granulocytes and apoptotic cleaved caspase (Casp)-3^+^ cells immunohistochemically, additional sections were used. A citrate buffer was used to demask antigens, and the unspecific binding sites were then blocked with goat serum. Cells were stained by incubating them with either a polyclonal rabbit MPO antibody (1:100; Abcam, Cambridge, UK) or a monoclonal rabbit-anti-mouse Casp-3 antibody (1:100; Cell Signaling Technology, Danvers, MA, USA) as the first antibodies, followed by a biotinylated goat anti-rabbit IgG antibody (ready-to-use; Abcam) as the second antibody. Peroxidase-labeled streptavidin (ready-to-use; Abcam) was used for the detection of the biotinylated antibody. 3-amino-9-ethylcarbazole (Abcam) was used as a chromogen. Mayer’s hemalum (Merck, Darmstadt, Germany) was used for the counterstaining. Within two randomized HPF selected in the flap’s proximal zone and the transition zone. 

### 2.7. Statistical Analysis

All data were tested for normal distribution and equal variance. Subsequently, differences between the two experimental groups were assessed by means of the unpaired Student’s *t*-test (GraphPad Prism 9; GraphPad Software, San Diego, CA, USA). A Mann–Whitney rank sum test was applied if non-parametric data were detected. All given values are stated as the means ± standard error of the mean (SEM). A statistically significant difference was accepted for a value of *p* < 0.05.

## 3. Results

### 3.1. Intravital Fluorescence Microscopy

**Figure 2:**
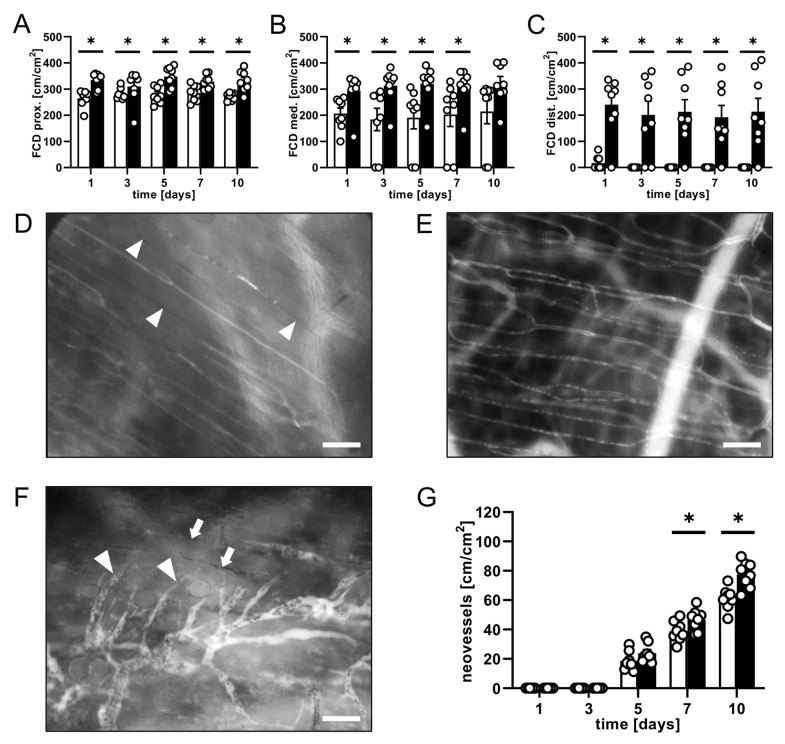
(**A**–**C**) FCD [cm/cm^2^] in the proximal (**A**), medial (**B**), and distal zone (**C**) of flaps in MVF-injected mice (black bars, *n* = 8) and vehicle-injected controls (white bars, *n* = 8) on days 1, 3, 5, 7, and 10 after flap elevation, as assessed by intravital fluorescence microscopy and computer-assisted image analysis. Means ± SEM (white circles = individual data points). * *p* < 0.05 vs. control. (**D**,**E**) Intravital fluorescent microscopic images of the capillary fields in the medial zone of flaps in a vehicle-injected control mouse (**D**); no longer perfused capillaries are marked by arrowheads and an MVF-injected mouse (**E**) on day 5 after flap elevation. Scale bars: 50 μm. (**F**) Intravital fluorescent microscopic image of angiogenic sprouts (marked by arrowheads) growing out of pre-existing microvessels. No longer perfused capillaries in the necrotic tissue adjacent to the transition zone can be detected (marked by arrows). Scale bar: 50 μm. (**G**) Neovessels [cm/cm^2^] in the transition zone of flaps in MVF-injected mice (black bars, *n* = 8) and vehicle-injected controls (white bars, *n* = 8) on days 1, 3, 5, 7, and 10 after flap elevation, as assessed by intravital fluorescence microscopy and computer-assisted image analysis. Means ± SEM (white circles = individual data points). * *p* < 0.05 vs. control.

Using repeated intravital fluorescence microscopy, the vascularization and overall survival of the musculocutaneous flaps within the dorsal skinfold chambers were assessed. A significantly lower necrosis rate of ~26–28% was detected in MVF-injected flaps when compared to the flaps of vehicle-injected controls, which exhibited a necrosis rate of ~42–51% over the course of the 10-day observation period (Figure 1B,C). A significantly higher FCD in all observed flap zones was associated with a markedly diminished flap necrosis rate (Figure 2A–E). In fact, in the proximal and medial zone of MVF-injected flaps, the FCD was ~300–350 cm/cm^2^. The distal zone still showed an FCD of ~200 cm/cm^2^ (Figure 2A–C). Contrastingly, the FCD in the proximal and medial zone of vehicle-injected flaps was markedly decreased (~200–280 cm/cm^2^). In the distal zone, it could only be measured on day 1 (~20 cm/cm^2^) (Figure 2A–C).

The vessel diameter and centerline RBC velocities in all examined vessel types (arterioles, capillaries, and venules) within the flaps were measured. From the values, the volumetric blood flow was calculated. It increased in all three vessel types in both groups throughout the 10-day observation period, as the flaps adjusted to the changed blood supply (Table 1). Moreover, arterioles and venules of MVF-injected flaps showed a tendency towards a higher volumetric blood flow when compared to the vessels in vehicle-injected flaps (Table 1).

The formation of new GFP^−^ blood vessels was analyzed in the transition zone between vital and necrotic flap tissue. In both groups, the flap tissue displayed characteristic changes, such as vessel dilation and irregular diameters in the capillary architecture, within this zone starting on day 5. In addition, angiogenic sprouts grew out of pre-existing microvessels (Figure 2F). Notably, the neovessel density was significantly higher in flaps of MVF-injected mice on days 7 and 10 when compared to that of flaps in control animals (Figure 2G).

**Figure 3:**
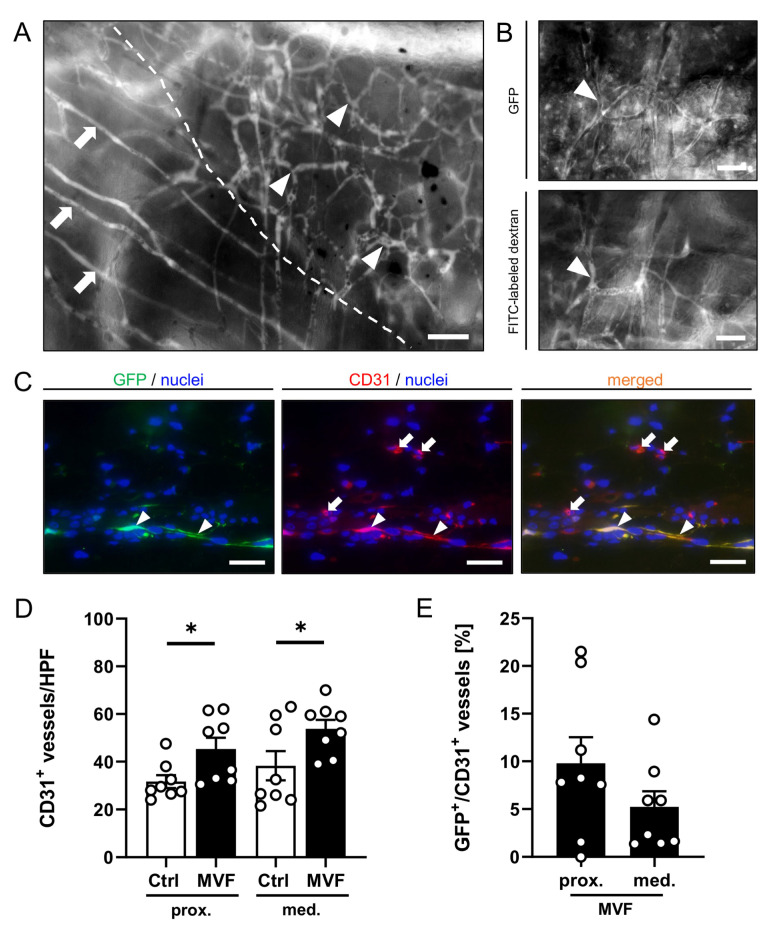
(**A**) Intravital fluorescent microscopic images of an MVF cluster (border marked by broken line). The densely cross-linked and chaotic vessel architecture (marked by arrowheads) can be clearly differentiated from the straight and parallelly arranged muscle capillaries (marked by arrows). Scale bar: 50 μm. (**B**) The GFP fluorescence signal (marked by arrowheads) of the microvessels confirms their origin from the GFP^+^ fat donors. In addition, the presence of FITC-labeled dextran within the vessels indicates their blood perfusion. Scale bar: 50 μm. (**C**) Immunofluorescent CD31/GFP stainings of MVF-injected flap tissue on day 10. CD31^+^/GFP^+^ microvessels (marked by arrowheads) originating from the injected MVF can be distinguished from the CD31^+^/GFP^−^ host microvasculature of the flap (marked by arrows). Scale bars: 25 μm. (**D**) CD31^+^ microvessels/HPF in the proximal and medial zone of flaps in MVF-injected animals (MVF, black bars, *n* = 8) and vehicle-injected controls (Ctrl, white bars, *n* = 8). Means ± SEM (white circles = individual data points). * *p* < 0.05 vs. control. (**E**): GFP^+^/CD31^+^ microvessels [% of all CD31^+^ microvessels] in the proximal and medial zone of MVF-injected flaps (black bars, *n* = 8). Means ± SEM (white circles = individual data points).

In addition, MVF clusters could be detected within the flap tissue throughout the observation period. Similar to the newly formed microvessels in the transition zone, their densely cross-linked and chaotic vessel architecture differed from the straight and parallelly arranged muscle capillaries (Figure 3A). Moreover, the emitted GFP-fluorescence signal confirmed their origin from the GFP^+^ donor mice (Figure 3B).

### 3.2. Histological and Immunohistochemical Analysis

Additional histological and immunohistochemical analyses were performed at the end of the in vivo experiments to examine morphological changes induced by ischemia within the flap tissue. HE-stained sections were used to identify the transition zone between the proximal and distal flap tissue. The distal zone was completely necrotic and therefore excluded from further immunohistochemical analyses.

In both the proximal and medial zone, a significantly higher number of CD31^+^ microvessels could be detected in MVF-injected flaps when compared to vehicle-injected controls (Figure 3D). About 5–10% of these microvessels were GFP^+^ and, thus, directly originated from the injected MVF (Figure 3E).

**Figure 4:**
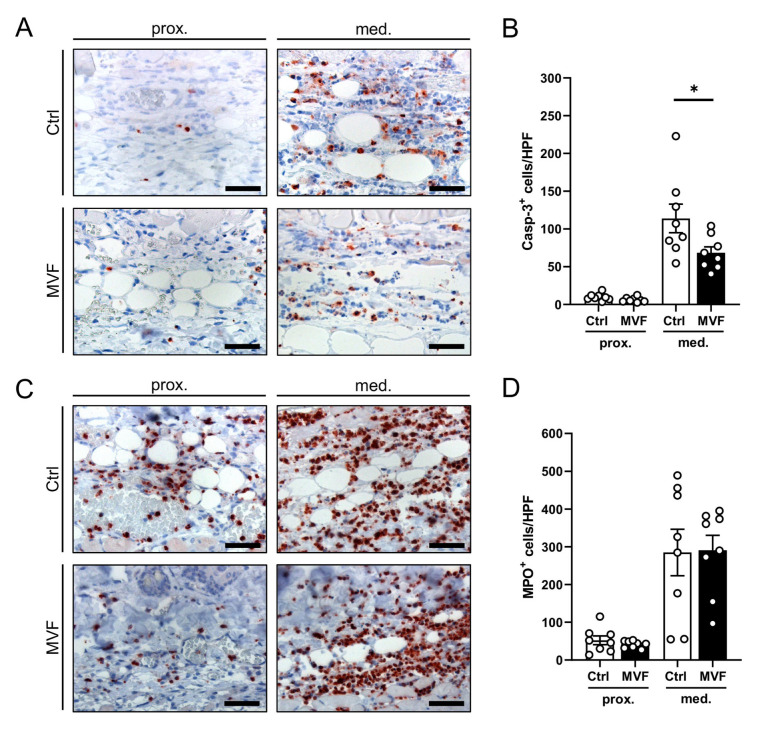
(**A**) Immunohistochemical tissue sections of the proximal and medial zone in flaps of a vehicle-injected control mouse and an MVF-injected mouse 10 days after flap elevation. The sections were stained with an antibody against Casp-3 as a marker for apoptosis. Scale bars: 50 µm. (**B**) Casp-3^+^ cells/HPF in the proximal and medial zone of flaps in MVF-injected mice (MVF, black bars, *n* = 8) and vehicle-injected controls (Ctrl, white bars, *n* = 8) 10 days after flap elevation, as assessed by immunohistochemistry. Means ± SEM (white circles = individual data points). * *p* < 0.05 vs. control. (**C**) Immunohistochemical tissue sections of the proximal and medial zone in flaps of a vehicle-injected control mouse and an MVF-injected mouse 10 days after flap elevation. The sections were stained with an antibody against MPO as a neutrophilic granulocyte marker. Scale bars: 50 µm. (**D**) MPO^+^ cells/HPF in the proximal and medial zone of flaps in MVF-injected mice (MVF, black bars, *n* = 8) and vehicle-injected controls (Ctrl, white bars, *n* = 8) 10 days after flap elevation, as assessed by immunohistochemistry. Means ± SEM (white circles = individual data points).

Apoptotic cells were identified using immunohistochemical Casp-3 staining. In both groups, the proximal vital zone of the flaps contained only a few apoptotic cells (Figure 4A,B). In contrast, apoptotic cell death was markedly increased in the medial transition zone. However, the number of Casp-3^+^ cells/HPF in this zone was significantly reduced in MVF-injected flaps when compared to vehicle-injected controls (Figure 4A,B). The additional identification of MPO^+^ neutrophilic granulocytes revealed a massive inflammatory cell invasion in the medial transition zone of both MVF-injected and vehicle-injected flaps without significant differences between the two groups (Figure 4C,D).

## 4. Discussion

The use of MVF is an established vascularization strategy in the field of tissue engineering. Due to their intact vessel morphology and functionality, MVF have shown a high potential to improve the incorporation of transplanted tissues and increase cell survival by rapidly assembling into blood-perfused networks connected to the host vasculature. As adipose tissue can be harvested from almost all patients in large quantities and the entire procedure for the isolation of MVF lasts only 30–45 min, a single-step intraoperative use of MVF in humans would be clinically feasible. In recent years, MVF from human adipose tissue have already been successfully used to create bioengineered tissues in preclinical studies [23,24]. Though in vivo use in patients has not been attempted yet, the transplantation of MVF represents a promising approach for the treatment of various pathologies associated with an insufficient tissue vascularization.

In this study, we evaluated the application of MVF for increasing the survival of random-pattern flaps. The present work is based on two pioneering studies in rats highlighting the potential of MVF to improve the outcome of flap surgery [4,5]. In line with these studies, we herein found that simple injections of freshly isolated MVF into murine random-pattern musculocutaneous flaps with pre-defined dimensions [17] effectively prevent necrosis of their distal tissue parts. Beyond that, we could demonstrate for the first time that MVF survive the transplantation procedure and reassemble over time into dense vascularization hotspots within the flap tissue.

We injected MVF from GFP^+^ donor mice into flaps within the dorsal skinfold chambers of GFP^−^ recipient animals. In combination with the technique of intravital fluorescence microscopy, this approach did not only enable us to follow the fate of the grafted MVF, but also to prove their final blood perfusion and, thus, their restored original functionality as vessel segments. The latter observation can be explained by the ability of MVF to rapidly reassemble into new microvascular networks, which develop interconnections to the surrounding host microvasculature [25]. Shepherd et al. [11] reported that this already occurs 24 h after MVF transplantation. Because flap necrosis usually demarcates after 3–5 days, this early boost in tissue vascularization seems to maintain nutritive perfusion during the initial critical period after flap elevation, resulting in a significantly lower rate of flap necrosis. In line with this assumption, we measured a markedly higher FCD in all flap zones of MVF-injected animals during the entire observation period when compared to vehicle-injected controls. Additional immunohistochemical analyses confirmed this finding with a significantly increased number of CD31^+^ microvessels in the proximal and medial flap zones after MVF injection. Most interestingly, only 5–10% of these microvessels were GFP^+^ and, thus, originated from the injected MVF. This indicates that MVF not only contribute as direct vascularization units to the nutritive perfusion of the flap tissue but also trigger other mechanisms mediating an improved flap survival.

It should be noted that MVF are a rich source of MSC [6,8,9,13]. Später et al. [13] already proved by flow cytometric analyses that isolated MVF contain 47.5 ± 4.7%, 7.1 ± 2.3%, and 9.2 ± 1% of cells expressing the mesenchymal stem cell markers CD29, CD90, and CD117, respectively. Furthermore, MSC have been shown in previous studies to exert a beneficial effect on the viability of flap tissue [26,27,28]. Uysal et al. [26] suggested that this effect may be partly traced back to their ability to differentiate into endothelial cells [26]. However, we speculate that in our study, an even more relevant mechanism may be the paracrine stimulation of angiogenesis by MVF. In previous studies, we could demonstrate that MVF secrete various pro-angiogenic factors, including VEGF [9,29]. Moreover, several studies could prevent flap necrosis by the application of VEGF [30,31,32,33]. This cytokine does not only stimulate blood vessel formation but also causes vessel dilation and maintains microperfusion via nitric oxide formation [34]. Although we did not perform additional analyses to confirm these findings in the present study, our novel results are consistent with the assumption that MVF exert strong paracrine effects on the microcirculation within the flap tissue. In fact, we detected a significantly higher number of newly formed CD31^+^ microvessels in the transition zone between vital and necrotic flap tissue of MVF-injected mice when compared to controls. In addition, we found a higher FCD in all zones of MVF-injected flaps over the course of the experiments.

Our immunohistochemical analyses further revealed a significantly reduced number of apoptotic cells within the medial transition zone of MVF-injected flaps when compared to vehicle-injected controls. This is an interesting finding, considering the fact that the inhibition of ischemia-induced apoptosis has been suggested to be effective in the prevention of flap necrosis [35,36,37]. We suggest that the herein observed anti-apoptotic effect of MVF injections is most probably caused by the improved nutritive perfusion of the flap tissue. However, we cannot exclude that MVF also directly inhibit apoptosis, which could be another explanation for their high regenerative potential in different experimental settings. This obvious assumption, which should be analyzed in detail in future studies, is supported by the fact that MVF contain MSC and macrophages, which both have the capability of secreting substantial amounts of anti-apoptotic cytokines, such as insulin-like growth factor-1 [38,39,40,41].

## 5. Conclusions

Taken together, the present study sheds more light on the beneficial effects of MVF on surgical flaps. Our experimental findings indicate that MVF protect ischemic musculocutaneous flap tissue from necrosis by improving nutritive tissue perfusion and suppressing apoptosis. Hence, the application of MVF may represent a promising strategy to enhance the success rates of flap surgery. In fact, it is conceivable that in a future clinical scenario, autologous MVF are rapidly harvested from the lipoaspirates of patients by means of automated separation systems, as they are already available for the isolation of stem cells or the generation of platelet-rich plasma [42,43], and then directly retransferred into freshly operated flaps. This intra-operative one-step approach, which would not be associated with additional extensive surgical interventions, could markedly contribute to reducing ischemic flap complications and the related patient morbidity.

## Figures and Tables

**Table 1:** Volumetric blood flow [pL/s] of vehicle-injected control mice (Ctrl; *n* = 8) and MVF-injected mice (MVF; *n* = 8) in arterioles, capillaries, and venules in the proximal, medial, and distal flap zones on days 1, 3, 5, 7, and 10 after flap elevation. The parameter was assessed by intravital fluorescence microscopy and computer-assisted image analysis. Means ± SEM. * *p* < 0.05 vs. control.

Volumetric Blood Flow [pL/s]		d1	d3	d5	d7	d10
Arterioles						
prox.	Ctrl	654 ± 108	871 ± 153	1081 ± 212	1150 ± 223	1503 ± 286
	MVF	773 ± 192	1273 ± 216	1371 ± 272	1741 ± 232	1962 ± 429
med.	Ctrl	367 ± 85	718 ± 168	736 ± 70	980 ± 160	1314 ± 247
	MVF	612 ± 131	983 ± 166	1108 ± 169	1530 ± 167 *	1885 ± 297
dist.	Ctrl	16 ± 3	-	-	-	-
	MVF	340 ± 88 *	765 ± 164	924 ± 228	1282 ± 312	1543 ± 299
Capillaries						
prox.	Ctrl	3 ± 0	4 ± 0	6 ± 0	5 ± 0	7 ± 0
	MVF	3 ± 0	4 ± 0	5 ± 0	5 ± 0	6 ± 1
med.	Ctrl	3 ± 0	4 ± 0	5 ± 0	5 ± 0	6 ± 0
	MVF	3 ± 0	5 ± 0	5 ± 0	6 ± 0	6 ± 1
dist.	Ctrl	0 ± 0	-	-	-	-
	MVF	2 ± 0 *	8 ± 5	4 ± 0	5 ± 0	6 ± 1
Venules						
prox.	Ctrl	518 ± 99	758 ± 160	1300 ± 279	1738 ± 468	1929 ± 782
	MVF	572 ± 76	1027 ± 197	1093 ± 213	2081 ± 640	1500 ± 368
med.	Ctrl	272 ± 88	711 ± 182	908 ± 234	1348 ± 251	1504 ± 291
	MVF	458 ± 102	1034 ± 218	1549 ± 299	2190 ± 443	2232 ± 580
dist.	Ctrl	65 ± 38	-	-	-	-
	MVF	239 ± 31 *	877 ± 272	1123 ± 182	2053 ± 528	2205 ± 515

## Data Availability

All data can be obtained in this manuscript.
